# From Adsorbent to Photocatalyst: The Sensitization Effect of SnO_2_ Surface towards Dye Photodecomposition

**DOI:** 10.3390/molecules26237123

**Published:** 2021-11-25

**Authors:** Kinga Michalec, Anna Kusior

**Affiliations:** Faculty of Materials Science and Ceramics, AGH University of Science and Technology, 30-059 Kraków, Poland; akusior@agh.edu.pl

**Keywords:** heterostructure, SnO_2_, SnS_2_, photocatalysis, adsorption, sensitization, multi-core@shell

## Abstract

Semiconductor photocatalysis is considered one of the most promising technologies for water purification from toxic organic dyes. However, to reliably evaluate the possibility of using a given material as a photocatalyst, it is crucial to investigate not only the photocatalytic activity but also its affinity towards various dyes and reusability. In this work, we studied the adsorptive/photocatalytic properties of hollow-spherical raspberry-like SnO_2_ and its SnO_2_/SnS_2_ heterostructures that were obtained via a chemical conversion method using three different concentrations of a sulfide precursor (thioacetamide). The adsorptive/photocatalytic properties of the samples towards cationic rhodamine B (RhB) and anionic indigo carmine (IC) were analyzed using uncommon wall zeta potential measurements, hydrodynamic diameter studies, and adsorption/photodecomposition tests. Moreover, after conducting cyclic experiments, we investigated the (micro)structural changes of the reused photocatalysts by scanning electron microscopy and Fourier-transform infrared spectroscopy. The obtained results revealed that the sensitization of SnO_2_ resulted not only in the significantly enhanced photocatalytic performance of the heterostructures, but also completely changed their affinity towards dyes. Furthermore, despite the seemingly best photocatalytic performance, the sample with the highest SnS_2_ content was unstable due to its (micro)structure. This work demonstrates that dye adsorption/desorption processes may overlap the results of cyclic photodecomposition kinetics.

## 1. Introduction

With rapidly growing industrialization and urbanization, water contamination is becoming an increasing problem worldwide [1]. Effluents from the dyeing, textile, and printing industries, due to their toxicity and carcinogenicity, pose a serious threat to human health and the environment [2]. It is estimated that about 70,000–105,000 tons of dyes are released into the environment annually [3]. The problem of dye-contaminated water has been addressed using various methods, such as biological treatment, membrane filtration, sorption processes, ion exchange, coagulation-flocculation, and catalytic oxidation [2,4,5]. However, many of these methods do not decompose dyes, but only transfer them to other media, creating secondary pollution that needs post-treatment [4,5,6]. Therefore, semiconductor photocatalysis, as a green and sustainable technology, has attracted a lot of attention in recent years [5,6]. In this process, organic dyes can be decomposed into non-toxic compounds (CO_2_ and H_2_O) under solar radiation and ambient conditions [4].

Among various semiconducting materials, tin dioxide (SnO_2_) is a promising candidate for a photocatalyst due to its non-toxicity, stability, high oxidation ability (valence band edge at 3.80 V vs. NHE [7]), high electron mobility (∼100–200 cm^2^ V^−1^ s^−1^ [8]), chemical inertness, photocorrosion resistance, and relatively low cost [7,8,9]. However, its photocatalytic activity is limited due to a wide bandgap (3.6–3.8 eV [10]) and fast recombination of photogenerated charge carriers [9]. To date, various strategies have been applied to extend its absorption range to visible light, such as doping, self-doping, stoichiometry alteration, and the formation of solid solutions or heterojunctions [9,11,12,13]. The latter is a particularly promising approach, as heterostructures also allow better charge-carrier separation and the matching of semiconductors with appropriate band edge potentials for redox reactions [9]. Since photogenerated charge carriers are transferred between coupled materials, their interface structure is extremely important [14]. For instance, the interfacial defects may act as the recombination centers and, consequently, hinder the transfer efficiency [15]. Therefore, it is crucial to synthesize heterostructures with a tight interface to increase charge separation [14,15]. Compared to the physical mixing of semiconductors, the chemical conversion of template nanoparticles allows obtaining heterostructures with stronger interaction, better homogeneity, and reduced self-agglomeration [14,16].

Another important factor that affects the overall photocatalytic efficiency of heterostructures is the adsorption of dyes, as it is the first step in a photodecomposition process [4]. Thus, the adsorptive properties of materials should be thoroughly investigated. On the one hand, when dye molecules cannot be adsorbed on the surface of the photocatalyst, the photocatalytic process would not be effective. On the other hand, the complete adsorption of dye may result in hindered light absorption. Consequently, fewer carriers can be generated and participate in a photodecomposition process. Therefore, besides the material’s predispositions to be applied in photocatalysis, it may only turn out to be an adsorbent due to its surface state. In general, the interactions between a photocatalyst and a dye are strongly dependent on both the surface properties of the photocatalyst and the structure of the dye. Depending on the surface charge of a photocatalyst, which is connected with a solution pH, the material may adsorb more favorably cationic or anionic dyes as a result of the electrostatic attraction [2,4,17].

To study whether a material may be applied as a photocatalyst, it is also crucial to investigate its reusability [18]. To this end, cyclic photodecomposition tests are usually performed. However, various factors may affect the measured kinetics, such as surface changes, blocking of active sites, or adsorption of the residues after the reaction. Thus, it is also important to analyze the materials’ structural and surface properties of the reused photocatalysts to evaluate their stability.

Herein, we investigated the sensitization effect of SnO_2_ nanomaterials with visible light-active SnS_2_ on their adsorptive and photocatalytic properties towards cationic (rhodamine B, RhB) and anionic (indigo carmine, IC) dyes. The SnO_2_@SnS_2_ heterostructures were obtained via chemical conversion of hollow-spherical raspberry-like SnO_2_ nanoparticles using different concentrations of an SnS_2_ precursor, as reported in our previous paper [19]. This work aims to demonstrate the importance of performing adsorption studies to fit a photocatalytically active material to a pollutant before the analysis of photocatalytic properties. In the adsorption analysis, we applied uncommon wall zeta potential measurements. The second purpose was to investigate the relationship between the (micro)structural and adsorptive/photocatalytic properties of the prepared samples. The results revealed that SnO_2_ and its heterostructures are characterized by completely different affinities towards RhB and IC. Moreover, the sample with the highest SnS_2_ content, despite the seemingly best photocatalytic performance, showed unstable properties due to its disintegrated morphology. The sample with a smaller amount of SnS_2_, but with preserved morphology, exhibited stable adsorptive and photocatalytic properties. The obtained results demonstrate that due to the complexity of the adsorption and photocatalytic processes, it is not possible to unequivocally determine the stability of the photocatalyst only based on cyclic photocatalytic tests. These studies may overlap the effects of dye adsorption/desorption processes and microstructural changes in the material.

## 2. Results

### 2.1. Structural and Optical Properties

**Figure 1:**
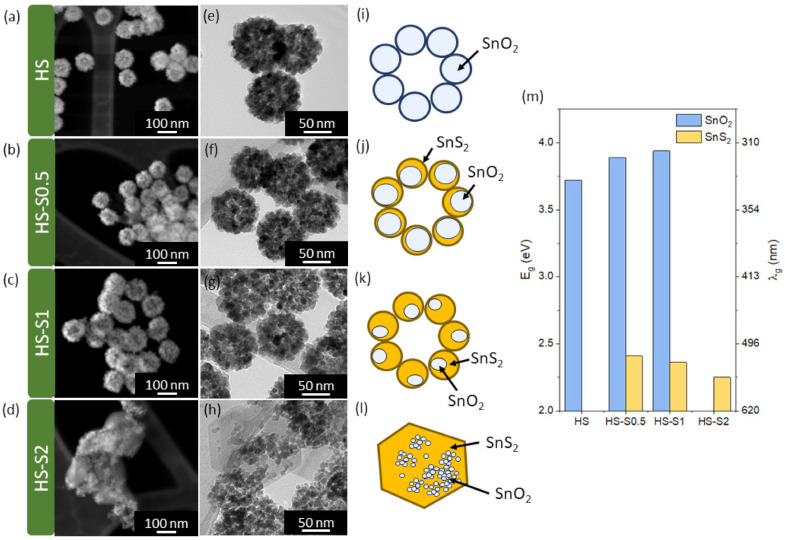
(**a**–**d**) STEM and (**e**–**h**) TEM images of the analyzed samples: (**a**,**e**) HS, (**b**,**f**) HS-S0.5, (**c**,**g**) HS-S1, (**d**,**h**) HS-S2; (**i**–**l**) schematic representation of the samples’ morphology; (**m**) diagram representing bandgap (E_g_) and absorption edge (λ_g_) values of the studied materials corresponding to SnO_2_ and SnS_2_.

The synthesis parameters of the analyzed materials, as well as their (micro)structural, surface, and optical properties, are presented in Table 1. The formation mechanism of SnO_2_/SnS_2_ heterostructures and in-depth characterization of their structure using X-ray diffraction (XRD), Raman spectroscopy, X-ray photoelectron spectroscopy (XPS), and high-resolution transmission electron microscopy (HRTEM) were reported in our previous work [19]. Briefly, the SnO_2_/SnS_2_ heterostructures were obtained via chemical conversion of hollow-spherical raspberry-like SnO_2_ nanomaterials (HS). In the syntheses, we used three different concentrations (Table 1) of a sulfide precursor (thioacetamide, TAA). Depending on the applied TAA:HS molar ratio, the synthesized samples exhibited different structural properties. For TAA:HS = 0.5 and TAA:HS = 1 (samples labeled HS-S0.5 and HS-S1, respectively), multi-core@shell SnO_2_@SnS_2_ nanomaterials were obtained. The reported multi-core@shell structure can be described as multiple single-core@shells that form a raspberry-like shape (see Figure 1j–k). The application of a higher TAA concentration resulted in an increased SnS_2_ content in the samples. For HS-S2 (TAA:HS = 2), however, the core morphology underwent disintegration.

Transmission and scanning transmission electron microscopy (TEM and STEM) images of the samples are presented in Figure 1a–h. The obtained results confirm that only in the case of HS-S2, the raspberry-like shape was completely disintegrated. It can be seen that this sample consists of larger grains surrounded by randomly distributed smaller elements. The measured grain size values of all samples are listed in Table 1. Moreover, since the analyzed samples are potential candidates for photocatalysts, their optical properties were examined. Based on the measurements of total reflectance spectra, the absorption edge (λ_g_) and bandgap values (E_g_) were determined (Table 1, Figure 1m). As expected, the HS sample can absorb only UV light and its bandgap (E_g_ = 3.7 eV) corresponds to SnO_2_ [20]. All HS-S samples, in turn, exhibit light absorption in the visible range. Bandgap values of about 2.3 eV can be assigned to SnS_2_ [14]. With an increasing SnS_2_ content, the absorption edge (λ_g_) from this component shifts towards longer wavelengths. Samples that preserve the raspberry-like shape (HS-S0.5, HS-S1) are characterized by E_g_ values corresponding to both the SnO_2_ and SnS_2_ components. In the case of HS-S2, in turn, only one bandgap from SnS_2_ was detected. This may indicate that the SnO_2_ core is covered with a thick layer of sulfide.

### 2.2. Adsorptive Properties

Adsorption of pollutants on the catalyst surface is the first step of a photodecomposition process. For this reason, the overall efficiency of a photocatalyst is strictly related to its surface properties. Depending on the net surface charge in aqueous solutions, a photocatalyst would have a higher affinity towards positively or negatively charged molecules. Therefore, zeta potential measurements were carried out (Supplementary Materials Figure S1) to characterize how the surface charge of the samples’ dispersions changes with pH. Based on the obtained results, all the samples characterize a negative surface charge over a wide pH range. It can therefore be assumed that they would more likely adsorb molecules with a net positive charge. The isoelectric point values (IEP) are listed in Table 1. Compared to HS, the IEP of the SnO_2_/SnS_2_ composites shift towards the acidic region. The lowest IEP value was recorded for the HS-S2 sample (IEP = 4.24) with the highest SnS_2_ content. Moreover, it is worth noting that HS has absolute zeta potential values (|ZP|) higher than 30 mV only in solutions with pH > 7.6. For SnO_2_/SnS_2_ heterostructures, in turn, such values were obtained in pH > 6.3 (HS-S0.5) and pH > 5.3 (HS-S1, HS-S2). Thus, the SnO_2_/SnS_2_ dispersions (HS-S0.5, HS-S1, and HS-S2) can be assumed to be stable in a wider pH range than pure SnO_2_ (HS) [13].

**Figure 2:**
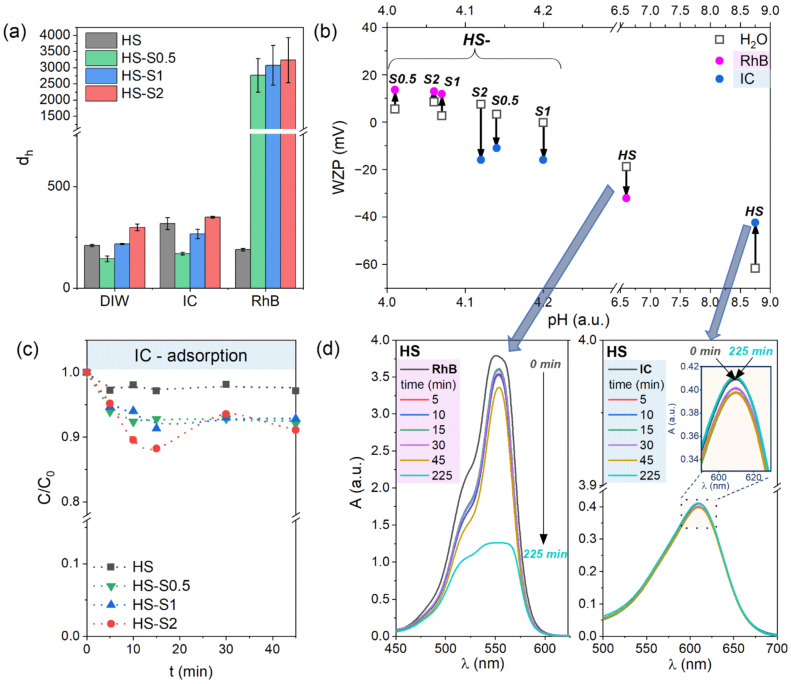
(**a**) Hydrodynamic diameter of the samples in deionized water (DIW), anionic indigo carmine (IC), and cationic rhodamine B (RhB); (**b**) diagram representing changes in wall zeta potential (WZP) values of the samples in rhodamine B (RhB) and indigo carmine (IC) compared to those obtained in aqueous solutions of the same pH; (**c**) adsorption kinetics of IC on the analyzed samples; (**d**) changes in the absorbance (A) of the dye solutions (RhB and IC) collected after various time intervals during adsorption tests for the HS sample.

For a better understanding of the nanoparticle dispersions’ properties, hydrodynamic diameter (d_h_) measurements were performed. This parameter allows characterizing the agglomeration tendency of nanopowders in aqueous solutions [21]. Moreover, the analysis of d_h_ values makes it possible to indirectly evaluate the adsorption behavior of powders towards various molecules, such as organic dyes [22]. Therefore, we conducted d_h_ measurements in deionized water (DIW) and aqueous solutions containing dyes with a net positive/negative charge. We chose rhodamine B (RhB) as a representative of cationic dyes and indigo carmine (IC) as anionic ones. The results are presented in Figure 2a. The hydrodynamic diameter values obtained in DIW are also listed in Table 1. It can be clearly seen that despite the similar grain size (Table 1), the HS-S0.5 sample has a lower d_h_ than the HS in DIW. This may indicate that HS nanoparticles have a higher tendency to agglomerate. Consequently, their reactivity might be reduced [21]. The obtained results are consistent with zeta potential measurements, which indicate that HS-S0.5 characterize higher |ZP| values. Both samples (HS and HS-S0.5) also show a similar tendency in anionic IC solutions. For HS-S1 and HS-S2, the d_h_ values are higher than in the case of HS despite the more stable suspensions of these compounds. Higher hydrodynamic diameter values may be connected with larger grain size and specific surface area of HS-S1 and HS-S2 (Figure S2). The presented BET results revealed that with an increasing SnS_2_ content in the samples, the specific surface area (S_BET_) of the obtained structures is higher. Therefore, it could be assumed that the HS-S2 sample would adsorb the highest amount of dyes.

For all samples, the average hydrodynamic diameter increase in IC solutions compared to DIW (318, 170, 267, and 349 nm for HS, HS-S0.5, HS-S1, and HS-S2, respectively). This increase may indicate the adsorption of dye molecules on the powders’ surface. Starkly different results were obtained for cationic rhodamine B solutions. In the case of HS, the average hydrodynamic diameter decreased compared to DIW (189 and 210 nm, respectively). Thus, RhB adsorption is highly likely to have affected the breakdown of HS agglomerates into smaller groups. For all SnO_2_/SnS_2_ heterostructures, in turn, the measured d_h_ values were approximately 10 times higher than in DIW and IC. On the one hand, this may indicate the strong affinity of these samples for cationic dyes and therefore confirm the assumption based on zeta potential measurements. On the other hand, these higher values may be related to the agglomeration of nanoparticles induced by the adsorption of RhB [22]. According to Talbot et al. [22], such agglomeration can affect the spatial distribution of dye molecules and, consequently, have an impact on adsorption equilibrium. This effect was also observed during our experiment. For each measurement of the SnO_2_/SnS_2_ samples in RhB, the obtained d_h_ values differed significantly from each other (see error bars in Figure 2a). Therefore, it can be assumed that the RhB adsorption equilibrium can be reached only in the case of the HS sample.

Wall zeta potential (WZP) is a parameter that can be used to study the adsorption kinetics of various soluble components [23]. When particles adsorb on the walls of the measuring vessel, its surface charge (wall zeta potential) changes. This measurement is very sensitive to any changes in the system, including the presence of additional ions or a change in the solution pH. Any modification of the analyzed material changes its ability to deposit. As a result of the dye adsorption on the adsorbent/photocatalyst’s surface, the target group of its application can be determined. Figure 2b presents how the WZP of the suspensions changes in RhB/IC compared to aqueous solutions of the same pH. For IC, it can be seen that the wall zeta potential of HS shifts towards higher values, while for HS-S composites (i.e., HS-S0.5, HS-S1, and HS-S2), it decreases. The opposite tendency was observed in the case of RhB solutions. Therefore, it can be assumed that HS characterizes adsorption behavior different from that of its heterostructures (HS-S). For the RhB solution, the WZP of HS shifts towards lower values, indicating that the dispersion is more stable. This is consistent with d_h_ measurements, which revealed that RhB adsorption caused the breakdown of agglomerates and therefore stabilized the suspension. In the case of the HS-S samples, in turn, WZP slightly shifted towards higher values. This suggests that the analyzed suspensions are not stable and have a tendency to agglomerate, which also confirmed the d_h_ results. It is also worth noting that the pH values of the suspensions in RhB suggest that HS-S powders have a net positive charge (see Figure S1), whereas HS is negatively charged. This implicates that HS-S samples repulse the molecules of cationic RhB, while HS attracts them. For the IC solution, the WZP of HS increased, indicating lower stability of the suspension. The reverse nature of changes for HS-S, in turn, suggests that their suspensions in IC are more stable. This is also consistent with zeta potential measurements. The HS-S suspensions in IC are positively charged, while HS characterize a net negative charge (Figure S1). Thus, HS-S attracts anionic IC molecules and HS repulses them. Based on the obtained results, it can therefore be assumed that the HS sample would characterize better adsorption properties towards RhB, while HS-S composites towards IC.

The adsorption properties of the powders were further analyzed by performing adsorption tests. Briefly, the suspension containing the dye solution and dispersed powder was continuously stirred in the dark at room temperature and under atmospheric pressure. At specific time intervals, the suspension was collected, and the powder was separated from the dye solution. Relative concentrations (C/C_0_) were determined from changes in absorbance intensity (A/A_0_) of powder-free solutions. The experimental setup is presented in Figure S3 (for adsorption tests, the light was turned off). Figure 2c presents the adsorption kinetics of IC on the studied samples. The obtained curves suggest that adsorption equilibrium was reached after 45 min of stirring in the dark for HS, HS-S0.5, and HS-S1. The amount of adsorbed dye was 3% for HS, while for HS-S0.5 and HS-S1 it was 7%. In the case of HS-S2, the process did not stabilize. Nevertheless, extending the experiment duration to approximately 4 h revealed a different adsorption behavior of the powders. For HS, the measured absorbance of the IC sample collected after 225 min (Figure 2d) suggests that the initially adsorbed amount of dye was almost completely desorbed. The results for HS-S0.5, HS-S1, and HS-S2 are presented in Figure S4. HS-S0.5 showed behavior similar to that of HS, however, the remaining amount of adsorbed dye was 4%. Only in the case of HS-S1, the absorbance of the sample did not change compared to that obtained after 45 min, indicating that the adsorption equilibrium was reached. The measured values for HS-S2 suggest that even after nearly 4 h the adsorption-desorption process did not stabilize. In the case of this powder, the amount of adsorbed IC was 19, 12, and 16% after 105, 165, and 225 min, respectively. The amounts of the adsorbed IC dye measured after different periods of time are presented in Figure S5a. It can be seen that with an increasing SnS_2_ content in the samples, they adsorbed a higher amount of IC after approximately 4 h of the experiment. This is consistent with the assumption based on the BET results. Analysis of the HS behavior towards RhB (Figure 2d), in turn, revealed that it adsorbed 67% of the dye after 225 min of stirring in the dark. Thus, the obtained results confirm the assumption based on WZP measurements regarding the better adsorption properties of HS and HS-S towards RhB and IC, respectively. Nevertheless, only for HS-S1, the IC adsorption-desorption process stabilized. This indicates that in addition to the materials’ specific surface area, their (micro)structural properties also have an influence on the adsorption behavior.

Since the adsorption equilibrium was not reached for HS-S2, unlike for HS-S1, we decided to investigate the morphology of these samples after the IC adsorption process (Figure S6). The presented SEM images reveal that the microstructure of HS-S2 underwent disintegration, while the hollow-spherical raspberry-like shape of HS-S1 remained unchanged. Therefore, it can be assumed that for the HS-S1 sample, the morphology of multi-core@shell SnO_2_@SnS_2_, as well as its surface properties (ZP and WZP) allow achieving the best adsorption properties towards IC.

### 2.3. Photocatalytic Properties

**Figure 3:**
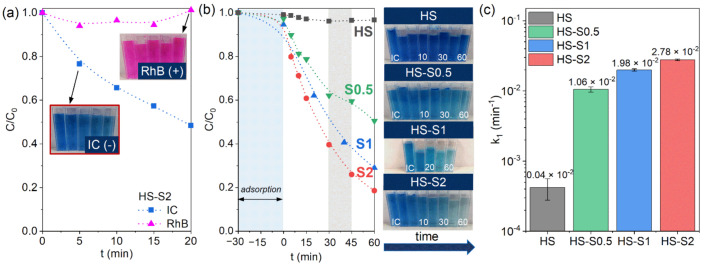
(**a**) Preliminary photocatalytic tests performed using SH-S2 to select a dye; (**b**) photodecomposition kinetics of IC obtained using HS, HS-S0.5, HS-S1, and HS-S2 under visible light illumination along with visual changes in dye concentration after various time intervals; (**c**) diagram representing calculated values of pseudo-first-order rate constants (k_1_).

The photocatalytic properties of the samples were investigated by performing dye decomposition tests under visible light illumination (see the experimental setup in Figure S3). Before illumination, the suspension containing the dye solution and the powder photocatalyst was stirred in the dark for 30 min. The samples were collected and analyzed in the same manner as for the dye adsorption tests. To reliably assess the photocatalytic performance of a catalyst, it is crucial to choose an appropriate dye for the tests. Based on the analysis of the adsorptive properties, we expected that HS-S composites would not decompose rhodamine B (RhB). Preliminary photocatalytic tests confirmed this assumption (Figure 3a). After 20 min of illumination, visible light-active HS-S2 decomposed approximately 50% of indigo carmine (IC), while the RhB concentration remained almost unchanged. This is consistent with the ZP, WZP, and d_h_ measurements, which revealed that the HS-S particles repulse RhB molecules and the suspensions are not stable. Therefore, the photocatalytic process cannot occur without effective adsorption. Based on the obtained results, IC was chosen for further photocatalytic tests.

Figure 3b presents the IC decomposition kinetics obtained under visible light illumination using HS, HS-S0.5, HS-S1, and HS-S2 as photocatalysts. The results reveal that the analyzed powders decomposed (and/or adsorbed) 3, 49, 71, and 81% of IC after 60 min of illumination, respectively. In the case of HS, its wide bandgap value (3.7 eV) does not allow it to decompose dye under visible light. Sensitization of SnO_2_ with visible light-active SnS_2_ resulted in a considerable enhancement of photocatalytic activity. In the case of HS-S0.5, it can be seen that the nature of the IC concentration changes differs from HS-S1 and HS-S2. For instance, between 30 min and 45 min of the process, only 3% of the dye was decomposed. Such behavior may be the result of IC desorption from the HS-S0.5 surface, which was confirmed by adsorption tests (Figure S4). It is also worth noting that despite the much higher SnS_2_ content in HS-S2 (32.9 wt.%) than in HS-S1 (11.6 wt.%), there is a slight difference in photocatalytic efficiency (10%) of the samples.

The investigation of the IC decomposition process pointed out that it follows pseudo-first-order kinetics according to the Langmuir-Hinshelwood model [24]. Therefore, the pseudo-first-order rate constants (k_1_) were determined as a slope of the linear plot of ln(C_0_/C) vs. irradiation time (Figure S5b). The calculated k_1_ values are presented in Figure 3c. It can be clearly seen that even applying a low SnS_2_ content (2.6 wt.% for HS-S0.5) considerably increases the photocatalytic efficiency of the systems. The highest k_1_ value was obtained for HS-S2 (2.78 × 10^−2^ min^−1^). However, in the case of this sample, the adsorption tests (Figure 2c, Figures S4 and S5a) revealed that this process did not stabilize over time. Therefore, the observed photocatalytic efficiency is a result of both adsorption and photocatalysis.

The effect of solution pH on the photocatalytic performance of HS-S2 was also examined, and the results are presented in Figure S7. The pH of the IC/HS-S2 solution without adjustment was 4. It can be seen that shifting pH towards the acidic region resulted in the deterioration of photocatalytic properties, while increasing pH to 5 led to better efficiency. A further increase in the pH solution, in turn, resulted in a reduced amount of decomposed dye. Despite a higher positive net charge of HS-S2 at pH = 3 (Figure S1), the increased concentration of H^+^ ions in the solution could cause anionic IC molecules to stay in the diffusion layer and thus hinder charge transfer from HS-S2 to IC [4]. Therefore, the measured photocatalytic efficiency was reduced. On the other hand, despite a net negative charge of HS-S2 at pH = 5 (Figure S1), the OH^−^ ions might push anionic IC molecules into the Stern layer and improve the charge transfer [4]. Therefore, the obtained efficiency was higher. A more negative net charge of HS-S2 at pH = 6 could increase the electrostatic repulsion of IC molecules and, thus, hinder the photocatalytic performance. To eliminate the influence of additional H^+^ and OH^−^ ions in the solution, further tests were performed without pH adjustment.

**Figure 4:**
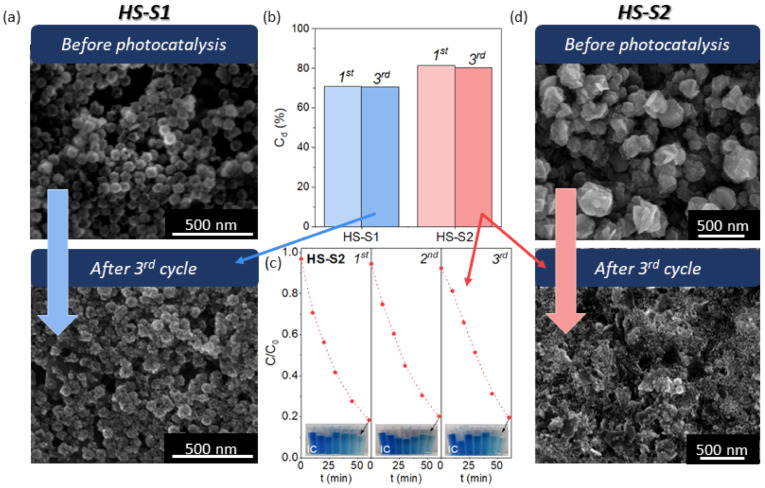
(**a**) SEM images of the HS-S1 sample before photocatalysis and after three cycles of indigo carmine (IC) photodecomposition under visible light illumination; (**b**) chart representing the amount of IC decomposed (C_d_) during the first and third cycle using HS-S1 and HS-S2 as photocatalysts; (**c**) cyclic photodecomposition kinetics of IC using HS-S2 as a photocatalyst; (**d**) SEM images representing microstructural changes of the HS-S2 sample after three cycles of IC photocatalytic decomposition.

To assess the stability of the photocatalysts, we performed cyclic photodecomposition tests of IC using the samples that showed the best photocatalytic performance (HS-S2, HS-S1). Briefly, after each cycle, the powder was collected and reused for the next one. The obtained photodecomposition kinetics and efficiencies (C_d_) after the first and third cycles are presented in Figure 4. For both samples, the measured photocatalytic efficiency only slightly decreased after the third run. The decrease in C_d_ value was minimally higher for HS-S2. Based on these measurements, it might seem that both catalysts are stable. Nevertheless, the results of cyclic photodecomposition experiments are not sufficient to evaluate photostability [18]. According to Chen et al. [18], various factors may affect the photocatalytic efficiency, such as adsorption of intermediates and surface changes. Therefore, we analyzed the morphology of the samples after performing cyclic tests (Figure 4). It was found that the microstructure of HS-S2 was completely disintegrated, whereas the shape of HS-S1 was preserved. The SEM images of HS-S1 collected after the first and second cycles are presented in Figure S6. It can be seen that after each cycle the raspberry-like morphology of multi-core@shell SnO_2_@SnS_2_ remained unchanged. The obtained SEM micrographs are consistent with those received after adsorption tests (Figure S6). They revealed that even IC adsorption (without photocatalysis) affects HS-S2 morphology. The microstructural disintegration of this sample might result in more sites available for dye adsorption. Therefore, despite the photocatalyst degradation, the measured efficiency after each cycle may appear unchanged. The BET results (Figure S2) revealed that the HS-S2 sample collected after the first IC photodecomposition cycle (labeled HS-S2-1st) characterized a slightly lower specific surface area than before conducting the cyclic experiments. However, as it can be seen from the SEM images (Figure 4 and Figure S6), the sample after performing the adsorption/photocatalytic tests consisted of a very small elements of about 30 nm in size. Therefore, as a result of high surface energy, they may have a strong tendency to agglomerate. Thus, the measured S_BET_ may in fact be the surface area of agglomerates and, consequently, it may be understated.

**Figure 5:**
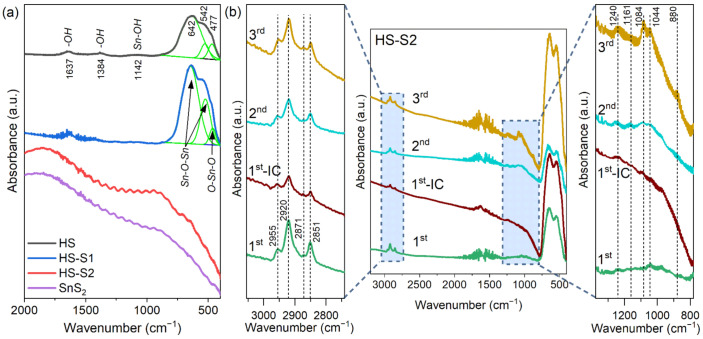
(**a**) FTIR spectra of the selected samples compared with pure SnS_2_; (**b**) FTIR spectra recorded for HS-S2 after performing cyclic photodecomposition tests (1st, 2nd, 3rd—the sample after 1, 2, and 3 cycles, respectively; 1st-IC—sample collected after the first cycle without washing in water/ethanol mixture).

To better understand the changes in the surface state of the HS-S2 sample, FTIR spectroscopy was applied. Figure 5a presents the results obtained for the as-synthesized HS, HS-S1, and HS-S2 samples, which were compared with pure SnS_2_. The FTIR spectra of HS-S2 after each photodecomposition cycle are shown in Figure 5b.

In the case of HS, the characteristic vibration bands for SnO_2_ at ca. 542 cm^−1^ and 642 cm^−1^ are visible (Figure 5a). These bands are typical for the Sn-O-Sn terminal oxygen vibrations mode. Moreover, the band at ca. 477 cm^−1^ can be attributed to the O–Sn–O bridge functional group of SnO_2_. The presence of additional absorption bands at 1384 and 1637 cm^−1^ is related to hydroxyl groups of molecular water adsorbed on the sample’s surface. The peak at ca. 1142 cm^−1^, in turn, can be considered as the Sn–OH stretching mode [25,26,27].

With an increasing SnS_2_ content in the samples, the vibration modes of tin dioxide are disappearing (Figure 5a). The recorded spectrum for HS-S2 is similar to that of pure SnS_2_. Therefore, it can be concluded that sulfide covers the SnO_2_ core with a thick layer, which was also confirmed by optical measurements (Figure 1m).

Figure 5b presents the results obtained for the HS-S2 sample collected after each photodecomposition cycle. Interestingly, after performing the first cycle (sample labeled 1st), the FTIR spectrum of HS-S2 no longer resembled that obtained for SnS_2_. This confirms the SEM observations, which revealed the disintegration of the HS-S2 microstructure. Consequently, the characteristic bands for SnO_2_ could be detected.

Furthermore, it should be noted that after each cycle the sample was rinsed with water/ethanol mixture (50/50 *v*/*v*) to remove unbound dye groups and dried at 25 °C under vacuum. For comparison purposes, one sample was dried immediately after collection without washing (the sample labeled 1st-IC). In each case, additional bands indicating the presence of residual dye were observed. The absorbance intensity of these bands is increasing after each cycle, which is the result of dye chemical bonding to the photocatalyst.

In the range of 800–1400 cm^−1^ are present bands that can be attributed to N–O (880 cm^−1^), S–O (1044 cm^−1^), C–O (1084 cm^−1^), and OC–O=H vibrations (1161 and 1240 cm^−1^ (δ_O–H_ + ν_C–O_)) [28,29]. These bands are characteristic of indigo carmine (IC). The vibration modes from 2800 to 3000 cm^−1^ can be assigned to the asymmetric (2851, 2871 cm^−1^) and symmetric (2920, 2955 cm^−1^) C–H stretching in CH_3_ and CH_2_ chains [28,30]. It is also worth noting that the 1st-IC sample shows some degree of fluorescence, which is characteristic of organic compounds (background elevation). Therefore, this provides evidence of the chemical bonding of the dye decomposition residues to the photocatalyst.

In addition to the (micro)structural and surface analysis of the reused powders, we collected the IC solution after performing the first photodecomposition cycle. The sample (labeled IC-HS-S2-1st) was analyzed using inductively coupled plasma optical emission spectroscopy (ICP-OES) to determine the presence of tin ions in the solution. The obtained results confirmed the degradation of HS-S2 during the photocatalysis. The measured Sn ions concentration for IC-HS-S2-1st was 0.2 mg/L (1.7 × 10^−6^ M), while for the reference sample containing IC solution before photocatalysis it was below the detection limit of the method (<0.001 mg/L). Based on the obtained results, it can be concluded that during photocatalysis the HS-S2 sample undergoes morphological and chemical changes in composition. The residues of the reaction products chemically bind to the surface of the grains, changing their properties. Moreover, the metal-ions from HS-S2 are leached into the IC solution. Consequently, the material changes its microstructure. This, in the case of its use as a photocatalytic material, is not a desirable phenomenon.

## 3. Discussion

Herein, we examined the adsorptive and photocatalytic properties of hollow-spherical raspberry-like SnO_2_ (HS) and its SnO_2_/SnS_2_ heterostructures (HS-S) that were obtained via chemical conversion using different concentrations of a sulfide precursor (TAA). Our previous studies [19] revealed that this process results in the formation of multi-core@shell systems. However, the amount of the precursor that can be involved in the ion-exchange reactions is limited. In the case of applying the highest TAA:HS molar ratio (2:1), the bonds that connected smaller individual elements forming the raspberry-like shape were weakened. Moreover, the measurements revealed that with an increasing TAA amount, additional redox reactions occurred in the system. Consequently, the morphology and phase composition of HS-S2 was changed. Apart from SnO_2_ and SnS_2_, this sample also contained sulfur and Sn_2_OSO_4_ (0.1 wt.% in total) [19].

Wang et al. [2] also examined the effect of TAA concentration on the photocatalytic properties of SnO_2_/SnS_2_ heterostructures towards K_2_Cr_2_O_7_ [20]. To cover SnO_2_ nanospheres, the authors applied the following molar ratios of TAA to SnO_2_: 1, 2, 3, 4, 5, 6, and 20. In the case of the highest TAA concentration, the well-defined morphology of porous nanospheres disappeared, and the sample comprised nanosheets with scattered nanoparticles. The authors reported that the 4:1 (TAA: SnO_2_) sample exhibited the best photocatalytic performance. From the 1:1 to 4:1 samples, with increasing SnS_2_ content, the efficiency was higher. The further increase resulted in decreased activity. The 5:1 sample, despite a much higher content of visible light-active SnS_2_ (49.2 wt.%) than the 4:1 one (19.5 wt.%), exhibited a much lower pseudo-first-order rate constant (0.0643 and 0.1859 min^−1^, respectively). This decrease in efficiency was ascribed to microstructural damage and hindered contact of the reactants with SnO_2_ during the photodecomposition process.

For our samples, increasing the SnS_2_ content resulted in enhanced adsorptive properties towards anionic indigo carmine (IC). Such behavior can be related to an increase in the specific surface area and changes in the surface charge. It might seem that the highest adsorption capacity of HS-S2 would contribute to improving the photocatalytic efficiency. Nevertheless, disintegrated morphology and the presence of additional phases from the SnO_2_-SnS_2_ system [19] caused undesirable changes in this material during photocatalysis. The IC photodecomposition residues chemically bound to the HS-S2 surface, changing its properties. Consequently, this material became unstable and therefore it would not meet the requirements for photocatalysts. The morphological changes and unstable adsorption properties were observed even without illumination (IC adsorption tests). HS-S1, in turn, preserved its raspberry-like shape after both adsorption and photocatalytic tests. Only in the case of this sample, the adsorption-desorption equilibrium of IC was reached. Thus, HS-S1 showed the best and most stable adsorption properties towards IC. Photocatalytic tests also revealed that despite much lower SnS_2_ content in HS-S1 (11.6 wt.%) than in HS-S2 (32.9%), there was a slight difference in their photocatalytic efficiency. This suggests that the multi-core@shell structure contributed to enhanced photocatalytic performance. Therefore, the microstructurally stable HS-S1 seems to be a promising photocatalyst for further examination.

In the case of HS, in turn, the analysis of adsorptive properties revealed that this sample characterized a completely different affinity for the dyes than its SnO_2_/SnS_2_ heterostructures. Because of its negative surface charge in the solutions of analyzed dyes, it can attract positively charged molecules of RhB. The HS sample adsorbed 67% of RhB after 225 min of the process. Therefore, despite its inactivity under visible light illumination, the obtained results suggest that HS could be further examined as an adsorbent material.

## 4. Materials and Methods

### 4.1. Chemicals

Sodium tin(IV) oxide trihydrate (98%, Na_2_SnO_3_·3H_2_O) was purchased from Alfa Aesar, Haverhill, MA, USA; anhydrous glucose (C_6_H_12_O_6_), thioacetamide (TAA, C_2_H_5_NS), hydrochloric acid (35–38%, HCl), sodium hydroxide (NaOH, 0.1 M), indigo carmine (IC, C_16_H_8_N_2_Na_2_O_8_S_2_), rhodamine B (RhB, C_28_H_31_ClN_2_O_3_), and ethanol (99.8%, C_2_H_5_OH) were from Avantor, Gliwice, Poland; and tin(IV) chloride pentahydrate (98%, SnCl_4_·5H_2_O) from Acros Organics, NJ, USA. All reagents were of analytical grade and used without further purification.

### 4.2. Synthesis

The synthesis procedure for obtaining hollow-spherical SnO_2_ nanomaterials (HS), as well as their composites with SnS_2_, was reported in our previous work [19]. Briefly, to obtain the HS sample, Na_2_SnO_3_·3H_2_O (8.0020 g) and glucose (21.6187 g) were dissolved in deionized water (200 mL). The solution was magnetically stirred for 15 min and then transferred to a Teflon-lined stainless-steel autoclave (BR-300, Berghof GmbH, Ravensburg, Germany) where it was heated at 140 °C for 180 min. Subsequently, the powder was centrifuged, washed several times in water/ethanol mixture (50/50 *v*/*v*), dried, and annealed in air at 500 °C for 180 min.

The SnO_2_/SnS_2_ composites were obtained via chemical conversion of the HS sample. In a typical experimental procedure, HS was dispersed in a solution containing deionized water (180 mL) and HCl (10 mL) using the ultrasonic treatment for 10 min. The concentration of HS in the solution was 0.058 mol/L. Subsequently, thioacetamide (TAA) was dissolved. The above mixture was placed in the same autoclave where it underwent hydrothermal treatment at 140 °C for 60 min. After centrifugation and washing in water/ethanol mixture, the powder was dried at 40 °C in a vacuum oven. Depending on the TAA concentration in the final solution, the prepared SnO_2_/SnS_2_ samples were labeled HS-S0.5 (0.029 mol/L), HS-S1 (0.058 mol/L), and HS-S2 (0.166 mol/L).

An SnS_2_ powder, which was used as a reference in the FTIR results analysis, was prepared similarly to the HS-S2 sample. The difference was that SnCl_4_·5H_2_O was applied instead of HS as a tin(IV) precursor.

### 4.3. Characterization

The morphology and chemical composition of the prepared materials were analyzed using Nova NanoSEM 200 (FEI Company, Hillsboro, OR, USA) scanning electron microscope (SEM) and Tecnai TF 20X-TWIN (FEI Company, Hillsboro, OR, USA) transmission electron microscope (TEM) equipped with an energy dispersive spectrometer (EDX) (FEI Company, Hillsboro, OR, USA). The TEM microscope also worked in the STEM mode with a HAADF image detector.

UV-Vis total reflectance spectra were recorded using a double-beam UV-ViS-NIR V-670 spectrophotometer (Jasco, Oklahoma City, OK, USA) with a 150 mm integrating sphere. The absorption edge (λ_g_) and bandgap (E_g_) values corresponding to direct (SnO_2_) and indirect (SnS_2_) forbidden transitions were estimated by applying the Kubelka-Munk function.

The specific surface area of the samples was determined on Nova 1200e (Quantachrome Instruments, Boynton Beach, FL, USA) using Brunauer-Emmett-Teller (BET) and Barrett-Joyner-Halenda (BJH) methods. Total pore area and volume values, pore size distributions, as well as BET surface area results, were reported in our previous work [19].

Isoelectric point (IEP) values were determined by measuring the zeta potential (ZP) of the samples’ dispersions in aqueous solutions with pH ranging from 3 to 10. ZP measurements were carried out using Zetasizer Pro (Malvern Panalytical Ltd., Malvern, UK).

FTIR absorption spectra of the samples were recorded on Excalibur 300 Series spectrometer (Digilab, Hopkinton, MA, USA), using the standard KBr pellet method. The scans were performed with a resolution of 2 cm^−1^ and over the range of 4000–400 cm^−1^.

The results obtained using X-ray diffraction (XRD), Raman spectroscopy, X-ray photoelectron spectroscopy (XPS), and high-resolution transmission electron microscopy (HRTEM) for the analyzed materials were described in detail in our previous work [19].

### 4.4. Investigation of Adsorptive Properties

The measurements of hydrodynamic diameter in deionized water (DIW) and dye solutions were performed using the dynamic light scattering (DLS) technique on Zetasizer Pro (Malvern Panalytical Ltd., Malvern, UK). Rhodamine B (RhB) and indigo carmine (IC) were applied as cationic and anionic dyes, respectively. The concentration of dye solutions was the same in all experiments (C_0_ = 5 × 10^−5^ mol/L). Wall zeta potential (WZP) values of the samples’ dispersions in DIW, RhB, and IC were determined via electrophoretic light scattering (ELS) technique using the same equipment.

The dye adsorption properties were also investigated by measuring the adsorption kinetics of RhB and IC. For each experiment, the powder (HS, HS-S0.5, HS-S1, or HS-S2) was added to a dye solution (C_0_ = 5 × 10^−5^ mol/L), and then the mixture was continuously stirred in a quartz beaker. In each case, the powder concentration was 1.25 g/L. The tests were carried out at room temperature and under atmospheric pressure. Moreover, the beaker containing the suspension of the powder photocatalyst and dye solution was placed in a cylindrical photoreactor (see Figure S3) with the lamps off, which was closed at the top with a cover. Thus, the measurements were conducted in the dark. At specific time intervals, the suspension was collected (4 mL) from the beaker using an MCE (mixed cellulose ester) syringe filter and centrifuged. Centrifugation was performed to ensure that the analyzed solutions did not contain powder residues. Thereafter, the absorbance of powder-free solutions was measured using a double-beam UV-ViS-NIR V-670 spectrophotometer (Jasco, Oklahoma City, OK, USA) in the range of 400–800 nm. The relative concentrations (C/C_0_) of RhB and IC solutions were determined from changes in the absorbance intensity (A/A_0_, where A_0_ is the maximum absorbance value of a dye solution before conducting the experiment).

### 4.5. Investigation of Photocatalytic Properties

Photocatalytic tests were carried out under visible light illumination using a cylindrical photoreactor (Figure S3) that comprised twelve Philips TL 8W/54–765 lamps and a quartz reaction vessel (beaker). For each experiment, the powder and dye concentration were the same as in the case of adsorption kinetics measurements. The tests were carried out at room temperature and under atmospheric pressure. Before illumination, the mixture was magnetically stirred for 30 min in the dark. During the photocatalytic process, the samples were collected at specific time intervals (using an MCE syringe filter and, thereafter, centrifugation) and analyzed in the same manner as for the dye adsorption experiments (the analysis of changes in the absorbance intensity). The pictures of the samples collected for the absorbance measurements are presented in Figure 3b. Analysis of the effect of solution pH on the photocatalytic properties was carried out using HCl and NaOH solutions (0.01 M) to adjust the pH of HS-S2/IC suspension.

The reusability of HS-S1 and HS-S2 samples was investigated by performing cyclic photodecomposition tests of indigo carmine (IC). After each 1 h cycle, the powder was separated from dye solution by centrifugation, washed several times in water/ethanol mixture (50/50 *v*/*v*), and dried at 25 °C under vacuum. Due to the mass loss of the photocatalyst, caused by collecting samples and centrifugation, the powder was weighed before each cycle and a proportionally smaller amount of dye solution was used. Changes in the morphology and surface properties of the samples after cyclic tests were analyzed by SEM and FTIR methods, respectively.

Inductively coupled plasma optical emission spectrometry (ICP-OES) analysis was performed using Optima 7300DV ICP-OES spectrometer (Perkin Elmer, Waltham, MA, USA).

## 5. Conclusions

In summary, the obtained results revealed that: (1) hollow-spherical raspberry-like SnO_2_ nanomaterials (HS) show affinity towards cationic rhodamine B (RhB) and, despite their inactivity under visible light illumination, they could be further examined as an adsorbent material for RhB removal; (2) sensitization of HS with SnS_2_ significantly enhances its adsorptive and photocatalytic properties towards anionic indigo carmine (IC); (3) performing adsorption studies to fit dye (anionic/cationic) to a photocatalyst is crucial before assessing its photocatalytic efficiency; and (4) the evaluation of a photocatalyst’s stability requires in-depth investigations of adsorption/desorption processes, photocatalytic tests, (micro)structural properties of reused photocatalysts, and the analysis of decomposed dye solution.

## Figures and Tables

**Table 1:** Synthesis parameters and characterization of (micro)structural, optical, and surface properties of the samples.

Sample	TAA: HS Molar Ratio	Reagent Concentration (mol/L)		Phase Composition (wt.%) [19]		Specific Surface Area (BJH Adsorption), S_BJH_	Grain Size, d (nm)	Hydrodynamic Diameter ^1^, d_h_ (nm)	Isoelectric Point, IEP (a.u.)	Absorption Edge, λ_g_ (nm)
		HS	TAA	SnO_2_	SnS_2_					
HS	0	0	0	100	0	34.018	78 ± 3	210 ± 6	4.61	333
HS-S0.5	0.5	0.058	0.029	97.4	2.6	37.502	79 ± 2	144 ± 14	4.47	319,515
HS-S1	1	0.058	0.058	88.4	11.6	44.564	87 ± 3	217 ± 3	4.45	315,525
HS-S2	2	0.058	0.166	67.0	32.9	54.297	167 ± 14	299 ± 17	4.24	551

^1^ The values obtained in deionized water (DIW).

## Data Availability

The data presented in this study are available on request from the corresponding author.

## Supplementary Materials

The following are available online, Figure S1: Zeta potential (ZP) of the analyzed samples (HS, HS-0.5, HS-S1, and HS-S2) in aqueous solutions as a function of pH, Figure S2: BET surface area plots, (b) chart representing the obtained specific surface area values (HS-S2-1st—a sample collected after the first cycle of IC photodecomposition), Figure S3: Schematic representation of the experimental setup for adsorption/photocatalytic measurements (for adsorption tests, the light was turned off), Figure S4: Changes in the absorbance of indigo carmine (IC) solutions collected after various time intervals during adsorption tests for: (a) HS-S0.5, (b) HS-S1, (c) HS-S2, Figure S5: (a) Chart representing the changes in the amount of the adsorbed dye (q_ads_) after different periods of time, (b) pseudo-first-order kinetic plots for the IC decomposition, Figure S6: SEM images representing the as-synthesized HS-S1 and HS-S2 samples, the powders collected after 45 min of indigo carmine (IC) adsorption process (HS-S1, HS-S2), and HS-S1 collected after the first/second cycle of IC photodecomposition, Figure S7: The effect of solution pH on the photocatalytic performance of HS-S2 S2 (C_d_—the amount of decomposed dye).

## Sample Availability

Samples of the compounds HS, HS-S0.5, HS-S1, and HS-S2 are available from the authors.

## References

[1] Jing L., Zhou W., Tian G., Fu H. (2013). Surface tuning for oxide-based nanomaterials as efficient photocatalysts. Chem. Soc. Rev..

[2] Gusain R., Gupta K., Joshi P., Khatri O.P. (2019). Adsorptive removal and photocatalytic degradation of organic pollutants using metal oxides and their composites: A comprehensive review. Adv. Colloid Interface Sci..

[3] Santhanarajan A.E., Sul W.J., Yoo K.J., Seong H.J., Kim H.G., Koh S.C. (2021). Metagenomic insight of a full scale eco-friendly treatment system of textile dye wastewater using bioaugmentation of the composite culture CES-1. Microorganisms.

[4] Chiu Y.H., Chang T.F.M., Chen C.Y., Sone M., Hsu Y.J. (2019). Mechanistic insights into photodegradation of organic dyes using heterostructure photocatalysts. Catalysts.

[5] Natarajan S., Bajaj H.C., Tayade R.J. (2018). Recent advances based on the synergetic effect of adsorption for removal of dyes from waste water using photocatalytic process. J. Environ. Sci..

[6] Li Y., Chen F., He R., Wang Y., Tang N., Thomas S., Pasquini D., Leo S.-Y., Gopakumar D.A. (2019). Chapter 24—Semiconductor Photocatalysis for Water Purification. Nanoscale Materials in Water Purification.

[7] Bandara J., Divarathne C.M., Nanayakkara S.D. (2004). Fabrication of n-p junction electrodes made of n-type SnO_2_ and p-type NiO for control of charge recombination in dye sensitized solar cells. Sol. Energy Mater. Sol. Cells.

[8] Chen H., Gu M., Pu X., Zhu J., Cheng L. (2016). Fabrication of SnO_2_@SnS_2_ heterostructure with enhanced visible light photocatalytic activity. Mater. Res. Express.

[9] Sun C., Yang J., Xu M., Cui Y., Ren W., Zhang J., Zhao H., Liang B. (2021). Recent intensification strategies of SnO_2_-based photocatalysts: A review. Chem. Eng. J..

[10] Abdelkarim O., Kaur J., Liu J., Navarro-Pardo F., Zarrin H., Yurtsever A., Bassioni G., Wang Z.M., Selopal G.S., Rosei F. (2020). Two-dimensional functionalized hexagonal boron nitride for quantum dot photoelectrochemical hydrogen generation. J. Mater. Chem. A.

[11] Li J., Wu N. (2015). Semiconductor-based photocatalysts and photoelectrochemical cells for solar fuel generation: A review. Catal. Sci. Technol..

[12] Wang H., Zhang L., Chen Z., Hu J., Li S., Wang Z., Liu J., Wang X. (2014). Semiconductor heterojunction photocatalysts: Design, construction, and photocatalytic performances. Chem. Soc. Rev..

[13] Kusior A., Zych L., Zakrzewska K., Radecka M. (2019). Photocatalytic activity of TiO_2_/SnO_2_ nanostructures with controlled dimensionality/complexity. Appl. Surf. Sci..

[14] Zhang Y.C., Du Z.N., Li K.W., Zhang M., Dionysiou D.D. (2011). High-performance visible-light-driven SnS_2_/SnO_2_ nanocomposite photocatalyst prepared via in situ hydrothermal oxidation of SnS_2_ nanoparticles. ACS Appl. Mater. Interfaces.

[15] Bai S., Xiong Y. (2015). Some recent developments in surface and interface design for photocatalytic and electrocatalytic hybrid structures. Chem. Commun..

[16] Anderson B.D., Tracy J.B. (2014). Nanoparticle conversion chemistry: Kirkendall effect, galvanic exchange, and anion exchange. Nanoscale.

[17] Kumar K.Y., Muralidhara H.B., Nayaka Y.A., Balasubramanyam J., Hanumanthappa H. (2013). Low-cost synthesis of metal oxide nanoparticles and their application in adsorption of commercial dye and heavy metal ion in aqueous solution. Powder Technol..

[18] Chen S., Huang D., Xu P., Xue W., Lei L., Cheng M., Wang R., Liu X., Deng R. (2020). Semiconductor-based photocatalysts for photocatalytic and photoelectrochemical water splitting: Will we stop with photocorrosion?. J. Mater. Chem. A.

[19] Michalec K., Kusior A., Mikuła A., Radecka M. (2021). New insights into the formation of multi-core–shell mesoporous SnO_2_@SnS_2_ nanostructures. Mater. Res. Lett..

[20] Wang S., Li G., Leng Z., Wang Y., Fang S., Wang J., Wei Y., Li L. (2019). Systematic optimization of promoters in trace SnS_2_ coating SnO_2_ nano-heterostructure for high performance Cr(VI) photoreduction. Appl. Surf. Sci..

[21] Suttiponparnit K., Jiang J., Sahu M., Suvachittanont S., Charinpanitkul T., Biswas P. (2011). Role of Surface Area, Primary Particle Size, and Crystal Phase on Titanium Dioxide Nanoparticle Dispersion Properties. Nanoscale Res. Lett..

[22] Talbot D., Queiros Campos J., Checa-Fernandez B.L., Marins J.A., Lomenech C., Hurel C., Godeau G.D., Raboisson-Michel M., Verger-Dubois G., Obeid L. (2021). Adsorption of Organic Dyes on Magnetic Iron Oxide Nanoparticles. Part I: Mechanisms and Adsorption-Induced Nanoparticle Agglomeration. ACS Omega.

[23] Reboiras M.D., Kaszuba M., Connah M.T., Jones M.N. (2001). Measurement of wall zeta potentials and their time-dependent changes due to adsorption processes: Liposome adsorption on glass. Langmuir.

[24] Guettaï N., Ait Amar H. (2005). Photocatalytic oxidation of methyl orange in presence of titanium dioxide in aqueous suspension. Part II: Kinetics study. Desalination.

[25] Pusawale S.N., Deshmukh P.R., Lokhande C.D. (2011). Chemical synthesis and characterization of hydrous tin oxide (SnO_2_:H_2_O) thin films. Bull. Mater. Sci..

[26] Majumder S. (2009). Synthesis and characterisation of SnO_2_ films obtained by a wet chemical process. Mater. Sci..

[27] Shaalan N.M., Hamad D., Abdel-Latief A.Y., Abdel-Rahim M.A. (2016). Preparation of quantum size of tin oxide: Structural and physical characterization. Prog. Nat. Sci. Mater. Int..

[28] Raji Y., Mechnou I., Yassine W., Kadri Z., Oumghar K., Cherkaoui O., Zyade S. (2020). Extraction of the natural indigo carmine pigment from the Isatis plant, characterization and dyeing of wool. IOP Conf. Ser. Mater. Sci. Eng..

[29] Mirabedini S.M., Moradian S., Scantlebury J.D., Thompson G.E. (2002). The role of interfacial layers on the performance of an epoxy/polyester powder coated aluminium alloy. Iran. Polym. J..

[30] Kumar D.S., Kumar P.S., Rajendran N.M., Anbuganapathi G. (2013). Compost maturity assessment using physicochemical, solid-state spectroscopy, and plant bioassay analysis. J. Agric. Food Chem..

