# Supplementary material for: From Adsorbent to Photocatalyst: The Sensitization Effect of SnO2 Surface towards Dye Photodecomposition
Source: Molecules. 2021 Nov 25;26(23):7123. doi: 10.3390/molecules26237123 (PMC8659128; doi:10.3390/molecules26237123)
Supplement: Supplementary file 1 [file molecules-26-07123-s001.zip › molecules-1479187-Supplementary.pdf]

Supplementary Materials

From adsorbent to photocatalyst: the sensitization effect of SnO_2 surface towards dye photodecomposition

Kinga Michalec* and Anna Kusior

Faculty of Materials Science and Ceramics, AGH University of Science and Technology, al. Mickiewicza 30, 30-059 Kraków, Poland

* Correspondence: kmichalec@agh.edu.pl

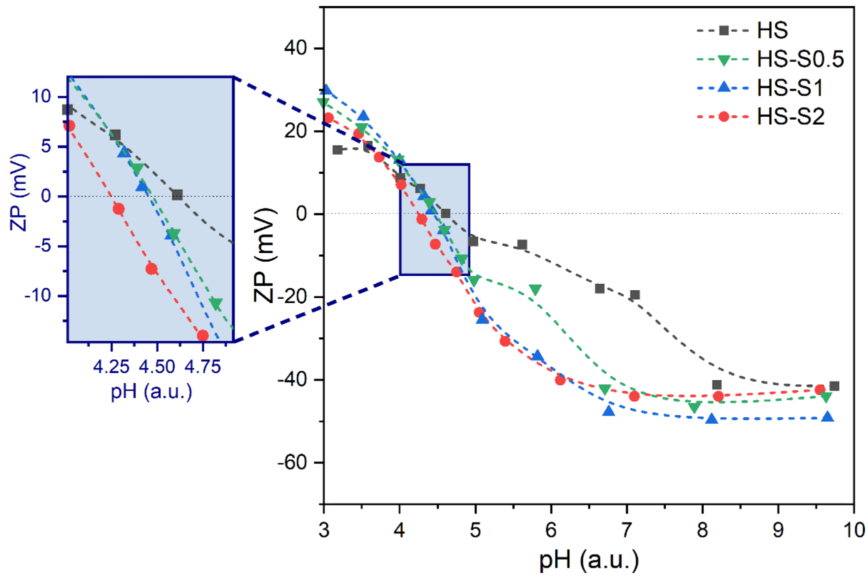

Figure S1. Zeta potential (ZP) of the analyzed samples (HS, HS-0.5, HS-S1, and HS-S2) in aqueous solutions as a function of pH.

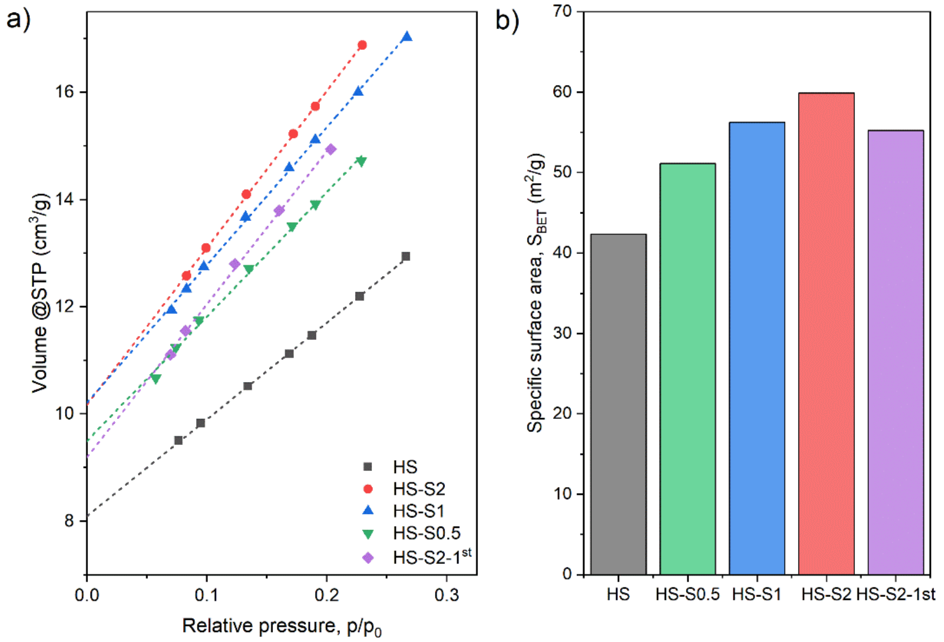

Figure S2. (a) BET surface area plots, (b) chart representing the obtained specific surface area values (HS-S2-1st – a sample collected after the first cycle of IC photodecomposition).

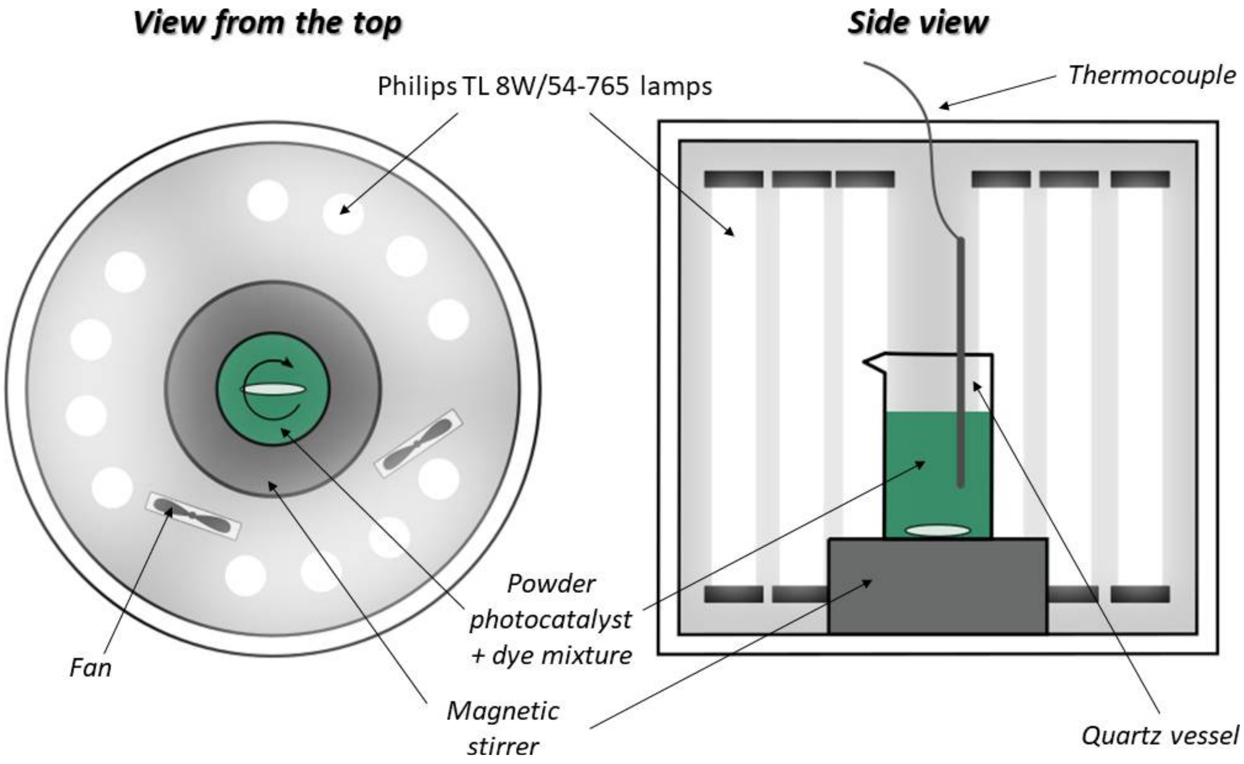

Figure S3. Schematic representation of the experimental setup for adsorption/photocatalytic measurements (for adsorption tests, the light was turned off).

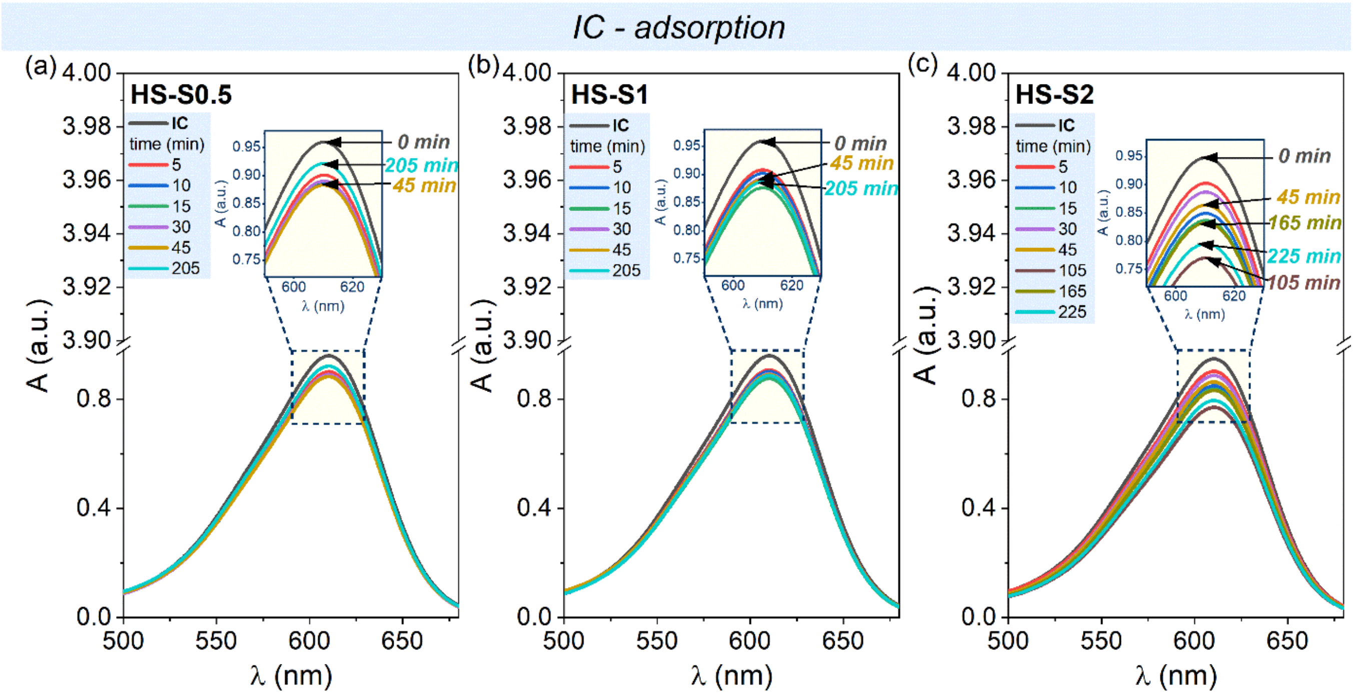

Figure S4. Changes in the absorbance of indigo carmine (IC) solutions collected after various time intervals during adsorption tests for: (a) HS-S0.5, (b) HS-S1, (c) HS-S2.

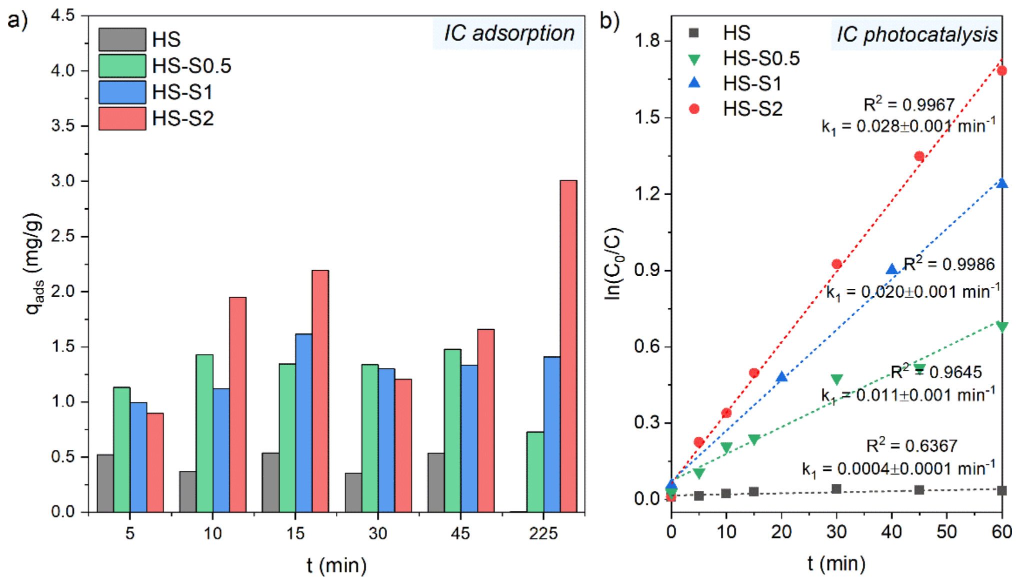

Figure S5. (a) Chart representing the changes in the amount of the adsorbed dye (q_{ads}) after different periods of time, (b) pseudo-first-order kinetic plots for the IC decomposition.

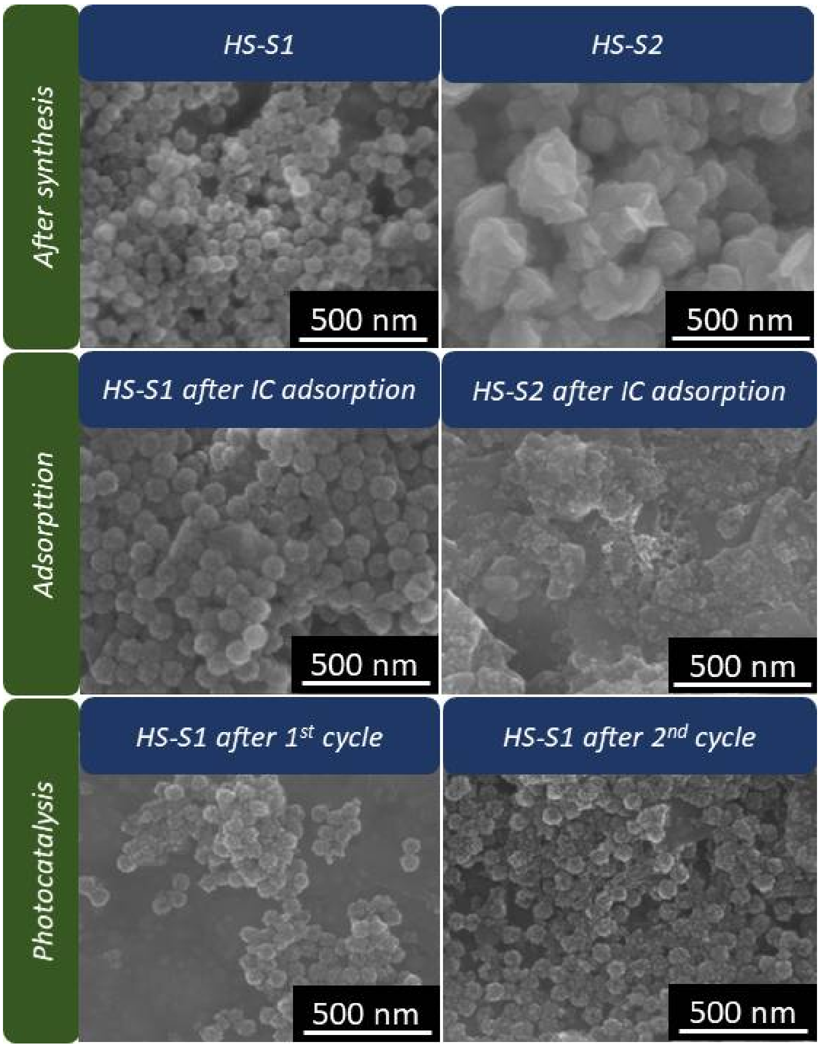

Figure S6. SEM images representing the as-synthesized HS-S1 and HS-S2 samples, the powders collected after 45 min of indigo carmine (IC) adsorption process (HS-S1, HS-S2), and HS-S1 collected after the first/second cycle of IC photodecomposition.

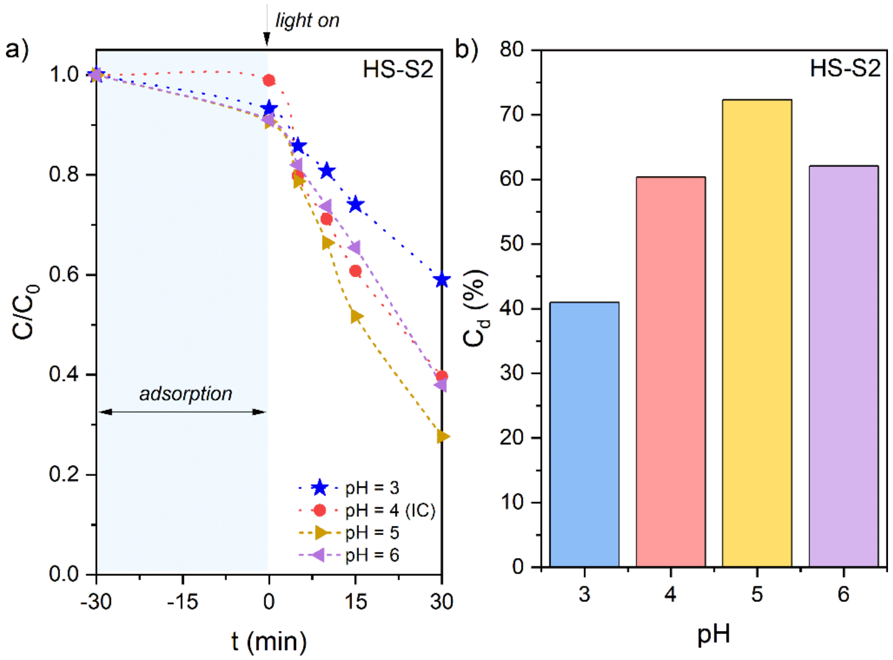

Figure S7. The effect of solution pH on the photocatalytic performance of HS-S2 S2 (C_d – the amount of decomposed dye).
